# Assessment of Attentional Processes in Patients with Anxiety-Depressive Disorders Using Virtual Reality

**DOI:** 10.3390/jpm11121341

**Published:** 2021-12-09

**Authors:** José A. Camacho-Conde, Leire Legarra, Vanesa M. Bolinches, Patricia Cano, Mónica Guasch, María Llanos-Torres, Vanessa Serret, Miguel Mejías, Gema Climent

**Affiliations:** 1 Department of Evolutionary and Educational Psychology, University of Granada, 51001 Ceuta, Spain; 2 Giunti-Nesplora Technology and Behavior, 20009 San Sebastian, Spain; leire#@nesplora.net (L.L.); mejias@nesplora.net (M.M.); climent230@nesplora.net (G.C.); 3 Hospitaller Sisters, Benito Menni Mental Healthcare Complex, 08830 Sant Boi de Llobregat, Spain; bolinches#@hospitalarias.es (V.M.B.); serret@hospitalarias.es (V.S.); 4 Hospitaller Sisters, Sacred Heart Hospital, 08760 Martorell, Spain; patricia#@hospitalarias.es; 5 Hospitaller Sisters, Vila de Gràcia-Cibeles Adult Mental Health Center, 08037 Barcelona, Spain; guasch#@gmail.com; 6 Hospitaller Sisters, Mother of God of Mercy Hospital, 08042 Barcelona, Spain; llanos@hospitalarias.es

**Keywords:** adults, anxious-depressive disorder, attention, continuous performance test, virtual reality

## Abstract

To characterize the attention deficits in one-hundred-fifteen participants, comprising two types of clinical profiles (affective and anxiety disorder), through a test of continuous VR execution. Method: Three tests (i.e., Nesplora Aquarium, BDI, and STAI) were used to obtain a standardized measure of attention, as well as the existence and severity of depression and anxiety, respectively. Results: Significant differences (CI = 95%) were found between the control group and the group with depression, in variables related to the speed of visual processing (*p* = 0.008) in the absence of distractors (*p* = 0.041) and during the first dual execution task (*p* = 0.011). For scores related to sustained attention, patients with depression and those with anxiety did not differ from controls. Our results suggest attentional deficits in both clinical populations when performing a continuous performance test that involved the participation of the central executive system of working memory.

## 1. Introduction

About 10–20% of patients seek primary care medical consultation for an episode of depression or anxiety, and more than 50% of these patients suffer from a second depressive disorder or comorbid anxiety [1,2]. The presence of anxiety–depressive comorbidity substantially increases the use of healthcare services and is associated with greater chronicity, slower recovery, higher recurrence rates, and greater psychosocial disability [1]. Increased recognition of the high prevalence and negative psychosocial impact of depression and the comorbidity of anxiety disorder will lead to more effective treatment [3].

Affective disorders, and more specifically major depressive disorder, are characterized by a depressed mood and/or a loss of interest or pleasure for at least two weeks [4], which may be accompanied by sudden physical, motor, and cognitive changes, as defined in the Diagnostic and Statistical Manual of Mental Disorders, Fifth Edition (DSM-5) [5].

Various studies have identified a pattern of neurocircuit disruption across major psychiatric disorders in regions and networks key to adaptive emotional reactivity and regulation. More specifically, disruption corresponded prominently to the ‘salience’ network, the ventral striatal/ventromedial prefrontal ‘reward’ network, and the lateral orbitofrontal ‘non-reward’ network [6]. In addition to affective symptoms, it has also been found that people with major depression have a decrease in various cognitive processes [7], such as processing speed [8,9,10], sustained attention [11], executive functions [12,13,14,15,16], visual memory [17], working memory [18], verbal fluency [19], episodic memory [20], visuospatial memory [19], selective attention or inhibitory control [11,21], and attentional arousal [22], among others. In addition, the existence of two different patterns of attention-deficit in clinical depression has been suggested: some depressives have a disorder of inhibitor distraction, and others show abnormalities in the processing of resources [23].

Anxiety disorders are characterized, in general, by an excessive and difficult to control concern, which occurs for at least six months, in relation to one or more specific events, activities, or objects that are perceived as a danger. It is distinguished from fear, in that the latter is an adaptive emotion that usually appears in predictable and acute situations or dangers, while anxiety arises from unpredictable and prolonged dangers (e.g., substantial external pressure, environmental stress, negative future expectations). Anxiety usually appears as restlessness or a feeling of continuous nervousness, ease of fatigue, difficulties in concentration and abstraction, irritability, muscle tension, and sleep problems [5,24].

Anxiety, when there is significant somatic symptomatology—for example, more than merely cognitive anxiety—is also related to a reduction in cognitive abilities, where processing speed [25,26], sustained [27,28,29] and selective attention [30], working memory [31], executive functions [28], inhibitory control [32], switching [33], and attentional arousal [22] are affected.

Virtual reality (VR) is a tool that allows the evaluation of cognitive processes through tasks integrated within an environment and allows such evaluation to be carried out in a more realistic way; relating the task performed to the challenges and difficulties that the evaluated person faces day to day and giving greater ecological validity to the obtained data [34]. In turn, this technology helps to maintain a high experimental control in the evaluation process, providing internal validity and a high degree of control over the variables of content, the delivery and measurement of stimuli, and the measurement and storage of responses in clinical evaluation or rehabilitation settings [35,36,37]. Allain et al. [38] suggest that, due to their peculiarities, VR tests can identify subtle deficits that are often undetected by traditional neuropsychological tests [39] and, therefore, VR allows a more accurate assessment. It is important to emphasize that the technology does not make the test, but merely provides the means for obtaining the above advantages. Moreover, the test must be validated, with a consistent theoretical base and correct psychometrics. Similarly to other neuropsychological tests performed on pen and paper or computerized, Nesplora Aquarium [40] is a validated VR test that can predict attention deficit hyperactivity disorder (ADHD) symptoms in adults and adolescents [41].

The objective of this study was to characterize the attention deficits in a sample comprising two types of clinical profiles (i.e., affective and anxiety disorder) using a test of continuous VR execution. Based on the variables provided by the test, we hypothesized that patients would have slower processing of stimuli than controls; people in the clinical groups would have attentional arousal, like the controls; deficits in selective attention would be observed in clinical patients relative to controls; and there would be deficits in sustained attention in the clinical patients relative to controls.

## 2. Materials and Methods

### 2.1. Participants

The analyzed clinical sample comprises 115 participants with a diagnosis of affective disorder or anxiety disorder using the DSM-5 criteria, who visited the mental health centers of Sisters Hospitallers in Sant Boi de Llobregat, Hospitalet, Martorell, and Barcelona and at the Torreblanca Psychology Center in Fuengirola (all in Spain). The inclusion criteria for the experimental group were (1) being between 16 and 69 years old; (2) having received a diagnosis of some type of anxiety or depressive disorder made by specialists (a neuropediatrician, clinical psychologist, or psychiatrist) according to DSM-5 criteria [5]; and (3) that the parents (in the case of minors <18 years old) and participants provided informed consent after reading the project’s information sheet. The inclusion criteria for the control group were: (1) 16 to 70 years old; (2) not diagnosed with attention deficit hyperactivity disorder (ADHD) by DSM-5; and (3) provided informed consent after reading the project’s information sheet.

In both groups, individuals were excluded if they met the following characteristics: (1) <16 years old or >70 years old; (2) presenting intellectual impairment, defined as an IQ <70; and (3) having a history of moderate or severe neurological disorders. After removing cases that did not meet the inclusion criteria, a final sample of 115 participants was obtained. In turn, for each clinical subgroup, a control group was generated from the normative study of Nesplora Aquarium, equivalent in age and sex.

The overall number of participants enrolled into the study was 230: 115 participants in the experimental group (depression, 38; anxiety, 77), and 115 in the control group (depression control, 38; anxiety control, 77). The participants were of both genders and aged 16–69 years (depression: mean (M) = 49.09; standard deviation (SD) = 12.05; depression control: M = 48.94; SD = 12.01; Mann–Whitney U test (U) = 622.5, *p* = 0.819; anxiety: M = 35.91; SD = 10.07; anxiety control: M = 36.07; SD = 10.33; U = 2123, *p* = 0.400). Table 1 shows some of the basic characteristics of the control and experimental groups.

Regarding the educational level of the experimental sample, primary studies (28%), secondary studies (19%), and university studies (53%) were observed. The control sample was compared at the same educational levels. The anxiety–depressive experimental group was heterogeneous, and the following main subtypes were observed: depression (major depressive disorder (27%), dysthymia (10%), mixed adaptive disorder (10%), non-specified depressive disorder (4%)) and anxiety (generalized anxiety disorder (9%), non-specified anxiety disorder (8%), agoraphobia (8%), obsessive-compulsive disorder (6%), panic disorder (6%), post-traumatic stress disorder (6%), bipolar disorder, social (5%), and phobia (1%)). The medication in both groups at the time of the assessment was as follows: depression (antidepressants (52%), hypnotics (18%), no medication (30%)) and anxiety (anxiolytics (79%), hypnotics (14%), non-medication (14%)).

The Ethical Committee of the University of the Basque Country approved the study and the data collection protocol for Research with Human Beings, CEISH. The study was carried out in accordance with the Code of Ethics of the World Medical Association (Declaration of Helsinki) for experiments with humans.

### 2.2. Procedure

All participants were recruited by consecutive sampling. Adults with anxiety-depressive disorders and controls were recruited through advertisements in universities, educational centers, the Nesplora Technology & Behavior social network, and on a psychology center website. The participants were not compensated for their participation; the only compensation was receiving their attentional report. The experimental sample was obtained from patients diagnosed with depression and anxiety who had been referred to the clinical psychology area of the assessment centers in this study. We chose the sample by taking all the patients who attended the centers for about 6 months. We considered controlling for their family-wise error rate. Regarding the estimated power of the study design, in order to determine the required sample size for the planned analyses, we made minor changes and adjustments at the beginning of the research to avoid bias, and we assigned a larger number of individuals to the anxiety group by collecting more diagnostic subtypes. The researchers planned to conduct a study with unequal groups, compute N as if we were using equal groups, and then compute the modified sample size. On the other hand, Giunti–Nesplora S. L directly selected the subjects of the control group. All participants were informed of the study procedures and asked for their consent to participate in the study. All sociodemographic variables were collected through the database of each center, following the guidelines dictated by Organic Law 15/1999 (December 13) on Protection of Personal Data [42].

The evaluations were carried out between November 2017 and May 2018. All tests were applied in all centers under similar conditions. The administration of Nesplora Aquarium and the collection of clinical data were carried out by an expert evaluator; that is, a neuropsychologist and a Giunti–Nesplora collaborator, who was adequately trained in standardized procedures for the administration of the test.

### 2.3. Measuring Instruments

Three tests (i.e., Nesplora Aquarium, Beck depression inventory (BDI), and state-trait anxiety inventory (STAI) were used to obtain a standardized measure of attention, as well as the existence and severity of depression and anxiety, respectively.

### 2.4. Nesplora Aquarium

Nesplora Aquarium [40] is a neuropsychological test that, through different continuous performance paradigms (CPT), evaluates attentional processes and working memory in people over 16 years of age and that has recently shown good psychometric properties related to its reliability and internal consistency values in a normative study, as well as being used in another series of studies [43,44,45,46,47,48,49]. The tool was found to have high internal consistency, and performance decreased with age, as would be expected. Unfortunately, the authors did not report any measures of construct and criterion validity. In addition, the difficulty and discrimination values of the items were acceptable [40]. The test is designed for the collection of reliable information on sustained and selective attention, visual and auditory attention, inhibitory control, reaction time and its deviation, perseverations, and switching (task change cost). It is an individual computerized test that lasts approximately 18 min and is performed through a VR system, consisting of a pair of glasses, a mobile phone, headphones, and a button. The system provides better visual and auditory immersion in the task than computerized CPTs. The tasks are based on CPT paradigms, but they are performed, as the name implies, in a virtual aquarium, where the person has to press a button every time he sees or hears certain fish or words, according to the instructions. Several elements of distraction in the environment are presented during tasks, to measure their effect on the motor activity and distraction of subjects. Instead of following a single model or theory, the test incorporates several scientific concepts. As a result, the Nesplora Aquarium is an integration of various models, which makes it a broad tool. The theoretical model of Sohlberg and Mateer [50,51] significantly aided the construction of this test, due to its contribution to the understanding of attentional processes. In addition, the Baddeley and Hitch [52] model was useful in designing the dual execution of the tasks included in Nesplora Aquarium [40]. The neuropsychological test is based on two CPT paradigms that are considered reliable measures of sustained attention [52]. Before starting the evaluation tasks, users perform a usability task and a learning task. As a result, (a) participants can familiarize themselves with the type of tasks they must complete later, and (b) participants do not become overexcited or anxious when using this type of technology. Nesplora Aquarium has an open response window; that is, the response time to the stimulus is not fixed. The time available to the person to respond to stimuli is adapted to their reaction time; if the response is fast, the next stimulus will appear earlier and if it is slow it will appear later, always with a time limit. This avoids keystroke errors that fall outside the expected window, and that can be counted as an error or success in the next item, when in reality it was a slow reaction time. For this reason, the execution time of the test can vary between 18 min and 23 or 24 min.

The tasks are explained as follows:

1. Usability task: This task enables the participant to become used to the virtual environment and have an opportunity to explore it and understand how the button works. The subject has to find and turn on four displays in the main room of the aquarium by putting a white dot seen in the center of the frame over each display and pressing the button.
2. Learning task training/learning task: This task consists of an AX or 1-back type text. The button must be pressed whenever the person sees the clownfish or hears the word ‘clownfish’, only if the previous fish or word was barbel. The learning task training has 20 items, and the learning task has 140 items. No neuropsychological evaluation data are produced from this task. The purpose of this first test is to train the participant and ensure they learn the stimuli.
3. Dual execution–Xno training/dual execution–Xno task: This is a Dual X_no or Dual No_go task. The person must press the button whenever a fish appears, or a word is heard, except when seeing the clownfish or hearing the word ‘barbel’; establishing a different target for visual and auditory channels. Training for Task 2 is made up of 20 items, and Task 2 has 140 items. The execution of this task is geared towards measuring selective attention, sustained attention, inhibitory control, and the central executive system, due to its dual component. Nevertheless, reaction time (RT) and variability of RT are also assessed.
4. Dual execution + i–Xno training/dual execution + i–Xno task: This is a Dual X_no or Dual No_go task with the interference of the previous task, due to the inversion of the target stimuli. The participant must press the button whenever they see a fish, or they hear a word (except when seeing the barbel or hearing the word ‘clownfish’); establishing a different target for visual and auditory channels. Training for Task 3 comprises 20 items, and Task 3 has 140 items. The execution of this task is geared toward measuring selective attention, sustained attention, inhibitory control, and the central executive system, due to its dual components. Nevertheless, reaction time (RT) and variability of RT are also assessed. In addition, through the inversion of the target stimuli, it is possible to evaluate the control of interference, both by switching capacity (cost of task change) and perseveration errors.

The tests provide the following measures:

- Omission errors: These errors occur when the participant does not press the button on the target stimulus. These types of errors are interpreted as a measure of the level of alertness, as well as the ability to selectively pay attention to the target stimulus. A standardized score above 60 points indicates inattention problems.
- Commission errors: These occur when the participant presses the button on a non-target stimulus. These errors represent an index of impulsivity or the ability to inhibit the response involved in selective attention processes. A standardized score above 60 points indicates impulsive behavior.
- Reaction time (RT): Specifically, this is the mean of the reaction time to correct answers. This measure indicates the average time elapsed from the presentation of the target stimulus until the button is pressed to respond. This measure reflects the participant’s response time. A standardized score above 60 is related to low processing speed.
- Variability of RT: Indicates the consistency of reaction time in correct answers. This measure is indicative of changes in sustained attention or fatigability during the task. A standardized score above 60 points indicates a fluctuation of attention during the test.
- Motor activity: This variable indicates the amount of movement of the head during the test, measured through the virtual reality glasses. This variable captures whether you have had excessive motor activity, and that you have stayed within the smaller frame, which represents the two rocks through which the fish appear, and the larger frame, which represents the angle of view from which you can see and perceive the visual stimuli to be responded to. This measure could be indicative of motor hyperactivity during the test. A standardized score above 60 points indicates hyperactive problems.
- Discrepancy: The discrepancy of correct answers between blocks. This score is obtained by comparing the hits in the first half of the task with those from the second half of the task. This gives additional information about the consistency of performance through each task. A standardized score above 60 points indicates minimal consistency in the performance of each task or fatigability during the tasks.
- Mean RT (reaction time)–commissions: This indicates the average time, from the stimulus appearing, until the button is pressed in incorrect presses (commissions). This measure gives us complementary information on commission errors. In this variable, a high score (low reaction time) is related to greater impulsivity and/or hyperactivity; while a low score (high reaction time) is considered a secondary measure of inattention [53]. Therefore, this variable provides explanatory information about the cause of commission errors.
- Switching: This score shows the difference between the number of hits in the last part of a task and the number of hits at the beginning of the next task. This variable provides information on the participant’s ability to adapt to a paradigm shift without their execution suffering. A standardized score above 60 points is a sign of difficulties changing tasks or switching.
- Switching RT–correct answers: This variable measures the difference between the reaction time of the hits in the last part of a task and the reaction time of the hits at the beginning of the next task. It provides information about the participant’s ability to adapt to the paradigm change without their reaction speed suffering. A standardized score above 60 points is a sign of difficulties switching tasks.
- Working memory: This variable is calculated from the correct items of the dual execution task and the dual execution task + i. These tasks involve different target stimuli for the visual and auditory channels. The parallel processing of both sensory modalities defines these exercises as dual execution tasks. These types of tasks are used for the evaluation of working memory. This index is interpreted inversely to the previous variables mentioned; in this sense, a standardized score of more than 60 points indicates good performance in the variable, because it is based on the number of successes.
- Perseveration: This type of error occurs in the dual execution task, with interference when responding to the task following the instructions of the previous task, in other words. This variable provides a measure of control of the participant’s retroactive interference. A standardized score above 60 points is interpreted as a deficit in cognitive flexibility.

### 2.5. BDI-II

The BDI-II [54] is a self-administered questionnaire that is used to detect the presence of depression; it is widely used, due to its brevity and ease of interpretation. It is composed of 21 multiple-answer questions and can be applied to people aged 13 years and older. The items are related to depressive symptoms, such as irritability, hopelessness, feelings of being punished, and physical symptoms related to the depressive state (e.g., fatigue, weight loss, sexual appetite).

### 2.6. STAI

STAI [55] is a self-administered questionnaire that is usually applied for the evaluation of anxiety and is composed of 40 items, of which 20 assess the anxiety state (present anxiety due to a specific event or situation), and the remaining 20 assess trait anxiety (level of anxiety as a personal characteristic). In addition to indicating the presence of anxiety and distinguishing it as an anxious state or an individual characteristic, it also provides information on its severity; in turn distinguishing it from depression and facilitating differential diagnosis.

### 2.7. Assessment Parameters

Processing speed is the time that a person requires to understand and react to a demand or mental task, whether visual, auditory, or motor. A slower processing speed means that it will take longer to perform activities such as reading, listening, engaging in conversations, or performing mathematical operations. It can affect other areas, such as executive functions, since people with a slow processing speed may have difficulty planning, making decisions, or setting goals, among other things. It is also related to the performance of simple or previously learned tasks or, in other words, the ability to process information automatically and quickly.

Processing speed is determined by measuring reaction time in the Nesplora Aquarium, which indicates the average time required to identify a target stimulus (visual or auditory) correctly. Shorter reaction times correspond to faster processing speeds. This measure was analyzed, both at a general level and in the different conditions presented by the test: auditory and visual items, conditions with and without distractors, and in the two dual-execution tasks performed.

Processing speed refers to the level of activation or attentional attention during the test and is a measure of the general level of alertness or surveillance used to carry out the requested task. It is usually in operation during the states of alertness, wakefulness and activation, while it is considered to be inactive in states of sleep or coma. An optimal level of arousal is necessary to be able to perform the tasks in the most efficient way possible, as the Yerkes–Dodson law explains, since, both, when the activation is insufficient and when it exceeds the limit at which the level of stress becomes excessive, the execution becomes deficient [56].

In Nesplora Aquarium, this construct is measured through omission errors, these being the errors that occur when the participant must press the target stimulus and does not. Higher omission errors correspond to a lower arousal during the test. This measure was analyzed both at a general level and in the different conditions presented by the test: auditory and visual items, conditions with and without distractors, and in the two dual-execution tasks performed.

*Selective attention* is the process in which a person selects an element, while ignoring the rest of the stimuli that occur or that are near the target. It is, in other words, the process of paying attention and selecting the important stimuli day to day; ignoring other objects or situations that are not relevant and may interfere with the task being carried out. Depending on the amount of effort or cognitive resources that the task requires, this type of attention can be consciously controlled or automatic.

More specifically, one aspect of selective attention has been examined: inhibitory control. This is a process of self-regulation, which allows inhibiting impulses and carrying out a more adequate and flexible action, aimed at achieving the objective in mind. For the evaluation of this process, the variables related to the number of commissions made in the task were analyzed. This measure refers to the number of times that the person presses the button before a stimulus that did not require pressing the button. This type of error determines whether there are difficulties in controlling or inhibiting a preponderant response and is a reliable measure of an individual’s impulsiveness, or if, on the other hand, the stimulus was not processed in time [57].

*Sustained attention* operates when a task requires maintaining attention on some task or stimulus, without departing from it, for a considerably prolonged period of time. It requires staying focused for long periods, even when the task is repetitive (e.g., reading, listening to an lecture) and/or there are distractors. In turn, it can be divided between the state of vigilance, in which the person is continually attentive to the appearance of the target, and concentration, in which the individual must remain focused on the task or stimulus.

Sustained attention can be measured and observed by the standard deviation of the different indices of the test. It is an indicator of how greatly the scores vary from the average. A high standard deviation may indicate that as the task progresses, the person becomes tired and increasingly needs more time to react, which increases the reaction time values of the beginning with respect to the end, showing a variability that, in the case that the performance or performance were continued, would not appear.

## 3. Results

Before applying the VR tool (Nesplora Aquarium), measures of depression and anxiety were used, namely the BDI and STAI. These measurements were used to differentiate the clinical sample. The participants’ BDI-II score indicated a moderate depression level (M = 29.39; SD = 8.28). These participants obtained a mean score of 28 (SD = 10.83) on the STAI-S (state subscale) and a mean score of 34 (SD = 11.12) on STAI-T (trait subscale). The participants’ state and trait anxiety were both ranked in the 80th-percentile (above average). The level of anxiety indicated a low anxiety level in both subscales. Based on the information extracted from the interview, self-report, observation, and the diagnostic criteria of the DSM-5, a diagnosis of anxious and depressive subtypes was confirmed.

The first cognitive process that was analyzed in this study was the processing speed.

### 3.1. Depression vs. Control and Anxiety vs. Control (Processing Speed)

A comparative analysis of the means of both groups and their respective controls was carried out using the non-parametric Mann–Whitney U test, obtaining the following results for the different processing speed variables analyzed (Table 2).

As indicated in Table 1, significant differences (CI = 95%) were found between the control group and the group with depression in the variables related to the speed of visual processing (U = 423.4; *p* = 0.008), in the absence of distractors (U = 492.3; *p* = 0.041), and during the first dual execution task (U = 441.1; *p* = 0.012). Table 1 shows that, unlike in the clinical depression group, no significant differences were observed in any of the variables related to the processing speed of patients with anxiety disorders, with respect to control subjects.

The second cognitive process that was analyzed in this study was attentional arousal.

### 3.2. Depression vs. Control and Anxiety vs. Control (Attentional Arousal)

A comparative analysis of the means of both groups and their respective controls was carried out using the non-parametric Mann–Whitney U test, obtaining the following results for the different variables of attentional arousal analyzed (Table 3).

As indicated in Table 2, significant differences (CI = 95%) were found between the control group and the group with depression, and between the control group and the group with anxiety, in all variables related to the level of attentional arousal, indicating a lower performance of the clinical group with depression in this function.

The third cognitive process that was analyzed in this study was inhibitory control.

### 3.3. Depression vs. Control and Anxiety vs. Control (Inhibitory Control)

A comparative analysis of the means of both groups and their respective controls was carried out using the non-parametric Mann–Whitney U test, obtaining the following results for the different inhibitory control variables analyzed (Table 4).

As the results of our analysis indicate, there were no significant differences in performance in these variables in any of the conditions evaluated. As with the sample with depression, patients with anxiety disorders did not differ from the controls in the scores referring to the capacity for inhibitory control of the response.

The fourth cognitive process that was analyzed in this study was sustained attention.

### 3.4. Depression vs. Control and Anxiety vs. Control (Sustained Attention)

A comparative analysis of the means of both groups and their respective controls was carried out using the non-parametric Mann–Whitney U test, obtaining the following results for the different sustained attention variables analyzed (Table 5).

Table 4 shows that there were no significant differences in performance in these variables in any of the conditions evaluated. As with the sample with depression, patients with anxiety disorders did not differ from controls in scores related to sustained attention.

## 4. Discussion

The results obtained in this study show the attentional deficits of two clinical populations when performing a continuous performance test with dual execution components, which involved the participation of the central executive system of the working memory. These results are in line with a recent normative study conducted with an adult population, with the objective of assessing attention and working memory [40]. In addition, attentional assessment can be useful to better understand the functional changes underlying the pathophysiologies of depression [58] and anxiety [59].

Previous studies have shown that the pulvinar nucleus of the thalamus plays important roles in contextual and multi-sensory processing, emotional response, and sustained attention [60,61,62,63]. In addition, the normal functioning of the pulvinar is impaired in mental disorders [6,64]. Affective disorders, specifically, in major depressive disorder (MDD), as reflected in abnormal functional connectivity (FC) in MDD, were characterized by abnormal FC between the amygdala, insula, anterior cingulate cortex (ACC), and prefrontal cortex (PFC). Cognitive impairment manifests as deficits in executive function, attention, memory, and rumination, primarily modulated by dysfunction between the fronto-parietal network and default mode network.

In the case of the group with affective disorders, a worse performance was observed in these scores related to the attentional arousal during the test; as was recently found in a population affected by ADHD [65]. Different studies have shown difficulties in updating working memory and in disengaging the focus on non-relevant information related to ruminant thinking in these patients [66,67,68,69]. In addition, in this clinical sample, significant deficits were observed in the processing speed of the stimuli [70], which could be related to the slowdown described both at the clinical level and in laboratory tests in these patients [19]. In addition, some studies have shown the effects of abnormal attention in the processing of irrelevant stimuli in major depressive disorder, as it is mediated by changes in effective connectivity within a distributed network of visual attention [58,71].

Regarding the group with anxiety disorders, our results describe only deficits in the scores related to attentional arousal or surveillance. Although attentional deficits in bias or hypervigilance disorders show that these patients had difficulties in maintaining an adequate attentional tone during the dual-execution tasks performed [72], a recent study has suggested that high anxiety reduces the attentional control and opens up the possibility that there is an inverse causality; that is, that low attention control increases anxiety [73]. Neuroimaging studies support an active role of the fronto-parietal attention network in sustained attention [74], but subcortical structures have also been proposed to be part of the network substrate of sustained attention [75]. Interestingly, impairment of sustained attention persists in patients with bipolar disorder [76] and major depressive disorder [77], even after achieving remission.

With respect to sustained attention and inhibitory control, our study did not find significant differences between each of the two clinical groups and their respective controls. However, anxiety has complex and powerful effects on cognitive performance. A recent study showed how anxiety can both influence attention and interfere with performance in a task facilitating information processing and behavioral responses. However, paradoxically, subjects with low attention control drive this effect. Therefore, anxiety increases the inhibitory control of prepotent responses, a mechanism that is adaptive under threat, and this effect is greater in subjects who rely more on the prepotent response; that is, those with low attention control [78].

It is known that the lateral posterior–pulvinar complex has extensive reciprocal connectivity with cortical areas including the visual and auditory cortices, with this circuit being fundamental to enhancing information processing and achieving contextual modulations in general [61].

Despite the extensive study of sustained attention at a behavioral level in both humans and animal models, surprisingly little is known about the circuit dynamics of sustained attention. Recently, we reported that a five-choice serial reaction time task (5-CSRTT) engaged the synchrony of oscillatory activity in the fronto-parietal network of the ferret [79,80]. Recently, Yu, Li, Stitt, Zhou, Sellers, and Frohlich [63] suggested that theta oscillation plays a central role in orchestrating thalamic signaling during sustained attention.

These results show that it is possible to use complex neuropsychological tests under virtual reality conditions to identify and describe the cognitive deficits that anxiety-depressive symptoms produce in these patients. A recent study of 2020, carried out by Camacho-Conde and Climent [43], showed the same results in adults with attention deficit hyperactivity disorder, in which it was shown that between 70 and 75% of adults with ADHD have a comorbid psychiatric diagnosis, the most common being a behavioral disorder, depression, anxiety, and borderline personality disorder, while, high levels of ADHD in adulthood are also related to psychotic disorders [81]. Deficits in executive functioning is a common feature of depression [12], and they also underlie depression-related difficulties in other cognitive domains, including execution/attention, memory, rumination, and visual/auditory [82,83]. Moreover, Luo et al. [84] found that executive function impairment is associated with increased FC between SN and FPN networks, and that reduced FC between the SCN and FPN surrounding the basal ganglia and thalamus might be associated with impaired top-down control exhibited in MDDs.

VR allows researchers to maintain a high level of experimental control. With VR, we can definitively eliminate evaluator bias, since the participant is protected from the halo effect, individual subjective effects, effects on performance, non-verbal messages, and the assessment of medium effects [85]. In VR tests, when the subject performs the test, the evaluator sees the subject’s execution, but does not intervene; the evaluator cannot make a change to the measurement, positively or negatively interfere with the result, or influence the subject through non-verbal language.

In this way, investigators must rely entirely on the test and its measurements [86]. The ability to integrate measures of movement adds value to this form of assessment, compared to traditional analogue tests and assessment scales. Therefore, this integrated way of studying cognitive and behavioral functioning is simply not obtainable with any other method [87]. In a traditional test, participants generate a mental representation of the object to be manipulated and then interact with it. However, with VR the object is presented virtually and spatial reasoning skills can be measured more directly. This, in turn, can lead to a higher predictive validity [88]. Rather than simply asking people to imagine spatial relationships, with VR it is possible to analyze their behavior when interacting with spatial problems [89].

Using VR to assess the cognitive processes of individuals with depression and anxiety might help overcome issues related to lack of motivation or increased fatigue during testing and, consequently, increase adherence. For example, some of the poor results on cognitive functioning obtained by people with depression can be explained by reduced motivation and fatigue during prolonged periods of testing [90,91].

VR tests are the form of test that can currently measure all the variables discussed: omissions, commissions, reaction time (like classic and computerized CPTs), a tendency to distraction, motor activity, and attentional focus deviation (only in VR). Furthermore, the user in the VR environment is totally isolated from his/her real world, but the VR optimizes the interactions in the real-world, while utilizing virtual contents [92]. Furthermore, a validated VR test (Nesplora Aquarium) is able to predict ADHD symptoms in adults and adolescents [41].

Nesplora Aquarium allowed us to significantly differentiate the dual-task execution between adult ADHD and characterize the attention deficits in a sample comprising two types of clinical profile (affective and anxiety disorders). Studies comparing adults with and without ADHD have produced imprecise results, as some studies have detected significant differences in performance in adults with ADHD, and other studies have found no such difference.

Weyandt et al. [93] investigated the performance of the Stroop test [94] with university students who were classified with significantly high or low ADHD symptoms, and found that these two groups did not differ significantly in their performance of this test. Walker et al. [95] investigated the performance of adults with ADHD relative to a psychiatric group and a control group, and reported that those with ADHD performed significantly worse on Conners’ CPT. However, the differences between ADHD and other psychiatric disorders were not significant, suggesting that the Nesplora Aquarium test can differentiate the healthy from the pathological, but might not be useful in differentiating clinical groups. We observed a deficit in motor and cognitive inhibition and problems in the control of emotions that directly affect social interaction.

These findings are not without limitations. Beyond the fact that the number of clinical samples was small, it should be noted that there was heterogeneity within them, which could have biased the results. It would, therefore, be convenient to filter by diagnosis, but a larger sample size is needed. In addition, for the present analysis, the severity of the symptoms collected through the BDI and STAI questionnaires, respectively, was not used to filter the evaluated sample; although the level of involvement in the groups was described. Our aim was to have specific studies of the main disorders that were previously described with larger samples. In fact, Nesplora Aquarium is currently being applied to various disorders in the population, as evidenced by the recent study by Camacho-Conde and Climent [43], conducted in adults with ADHD, to assess their attentional profile; a clinical case of a patient with PSTD [44]; a study based on the control of temporary disability due to common contingency in patients with minor psychiatric disorder [45]; and a recent study that examined the effectiveness of Nesplora Aquarium, evaluating participants with low and high symptoms of depression and anxiety [46]. Another limitation is the signs of cognitive deficits that are evident with aging, being more evident in subjects older than 50 years; we recognize that depressive symptoms can be predictors of visual memory deficits in middle age [96], and older anxious subjects displayed cognitive impairments in short-term memory; while older depressed patients showed executive dysfunction and more general cognitive impairments, not evident in older anxious subjects. [97]. In addition to affective symptoms, it has also been found that people with major depression have a decrease in various cognitive processes [7,98]. The difficulty in diagnostic assessment is particularly evident in older adults, because of the additional confusion created by age-related cognitive deficits [45].

We expect that distractibility will be an important part of our future work. Nesplora Aquarium includes multiple distractors, which, if enhanced, could be used to test how well adults maintain attention. However, if these distractors are over-emphasized, the task, which is already difficult, might become overly challenging. We found that those patients with depression responded differently to distracting stimuli, compared to those without depression. This VR result is also likely to translate into real-world settings [99]; life is full of distractions, and how one responds to these is surely linked to the ability to maintain attention when encountering distractors. These findings will be useful in the design of future studies and as an early warning to the patient, their family, and therapists when designing treatment.

## Figures and Tables

**Table 1:** Basic characteristics of the control and experimental groups (n = 230; excluded n = 14).

	Control				Clinical			
Gender	Male	35 (15%)	Female	80 (35%)	Male	35(15%)	Female	80 (35%)
Medium Age (years)		36.21 ± 22 (18–62)		41.66 ± 26.14 (16–69)		38.18 ± 21 (18–62)		42.66 ± 25 (16–66)
Inclusion features	16 to 70 years	35 (15%)	80 (35%)			35 (15%)		80 (35%)
	Not diagnosed ADHD	35 (15%)		80 (35%)		35 (15%)		80 (35%)
	Informed Consent	35 (15%)		80 (35%		35 (15%)		80 (35%)
Exclusion features	<16 to >70 years	1 (15%)		2 (35%)		2 (15%)		3 (35%)
	Intellectual impairment (IQ 103 < 70)	0 (0%)		0 (0%)		0 (0%)		0 (0%)
	Moderate/severe neurological disorders	2 (0.8%)		1 (0.4%)		1 (0.4%)		1 (0.4%)
	Diagnosed ADHD	1 (0.4%)		0 (0%)		0 (0%)		0 (0%)

**Table 2:** Contrast of means between groups with depression and anxiety and controls. Processing speed.

		Depression and Controls			Anxiety and Controls	
	U	z	*p*	U	z	*p*
Mean of the reaction time correct answers (MRTCA)	523.1	0.491	0.062	1824	−1.207	0.221
Visual right MRTCA	423.4	−1.554	0.008 *	1861	−1.234	0.267
Auditory MRTCA	547.3	−1.482	0.158	1784	−1.415	0.160
Distractor-affected items MRTCA	562.3	−1.383	0.171	1804	−1.383	0.188
Non-distractor-affected items MDTCA	492.3	−2.082	0.041 *	1861	−1.123	0.273
XnoDUALab_MRTCA	441.1	−2.227	0.012 *	1759	−2.332	0.138

Note: Mann-Whitney U test. For all tests, the alternative hypothesis specifies that the control group is less than the depression group. XnoDUALab_MRTCA = mean of reaction time of correct answers from block 2 of task XnoDUALab and correct responses from block 1 of task XnoDUALab; XnoDUALba_ MrTCA = mean of reaction time of correct answers from block 2 of task XnoDUALba and correct responses from block 1 of task XnoDUALba. * *p* < 0.05 ** *p* < 0.01.

**Table 3:** Contrast of means between groups with depression and anxiety and controls. Attentional arousal.

		Depression and Controls			Anxiety and Controls	
	U	z	*p*	U	z	*p*
Total omission errors	467.0	−2.471	0.016 *	1395	−4.107	0.002 **
Visual omission errors	421.5	−1.792	0.007 *	1310	−4.214	0.00 1 **
Auditory omission errors	499.0	−1.482	0.046 *	1474	−2.425	0.00 6 **
Distractor-affected items omission errors	472.5	−0.832	0.025 *	1509	−2.586	0.010 *
Non-distractor-affected items omission errors	463.0	−2.442	0.018 *	1390	−1.341	0.002 **
XnoDUALab_omissions errors_n	464.5	−2.319	0.020 *	1408	−1.532	0.002 **
XnoDUALba_omissions errors_n	459.2	−1.517	0.017 *	1465	−2.082	0.005 **

Note: Mann–Whitney U test. *Note.* For all tests, the alternative hypothesis specifies that the control group is less than the depression group. XnoDUALab__omissions errors_n = mean of reaction time of omissions errors from block 2 of task XnoDUALab and omissions errors from block 1 of task XnoDUALab; XnoDUALba__omissions errors_n = mean of reaction time of omissions errors from block 2 of task XnoDUALba and omissions errors from block 1 of task XnoDUALba. * p < 0.05 ** p < 0.01.

**Table 4:** Contrast of means between group with depression and anxiety and controls. Inhibitory control.

		Depression and Controls			Anxiety and Controls	
	U	z	*p*	U	z	*p*
Total commission errors	659.5	−0.592	0.519	1395	−0.530	0.719
Visual commission errors	621.0	−2.092	0.361	1310	−1.731	0.833
Distractor-affected items commission errors	672.5	−1.981	0.613	1474	−0.345	0.677
Distractor-affected items, mean of the reaction time correct answers	591.0	−1.152	0.249	1509	−0.603	0.541
Non-distractor-affected items commission errors canswers	661.5	−0.592	0.568	1390	−0.281	0.760
XnoDUALab_commissions errors_n	716.5	−0.364	0.789	1408	−1.794	0.701
XnoDUALba_comissions errors_n	644.0	−0.792	0.491	1465	−0.816	0.492

Note: Mann–Whitney U test. For all tests, the alternative hypothesis specifies that the control group is less than the depression group. XnoDUALab_ commissions errors_n = mean of reaction time of commissions errors from block 2 of task XnoDUALab and commissions errors from block 1 of task XnoDUALab; XnoDUALba_ commissions errors_n = mean of reaction time of commissions errors from block 2 of task XnoDUALba and commissions errors from block 1 of task XnoDUALba. * *p* < 0.05 ** *p* < 0.01.

**Table 5:** Contrast of means between groups with depression and anxiety and controls. Sustained attention.

		Depression andControls			Anxiety and Controls	
	U	z	*p*	U	z	*p*
Standard deviation of the reaction time correct answers (SDRTCA)	591.5	−1.102	0.277	1971	−0.530	0.781
Visual SDRTCA	614.5	−0.956	0.345	1725	−1.441	0.103
Auditory SDRTCA	507.1	−1.981	0.191	2114	−0.321	0.740
Distractor-affected items SDRTCA	620.3	−1.512	0.382	2052	−0.427	0.638
Non-distractor-affected items SDRTCA	609.5	−0.987	0.333	1947	−0.281	0.424
XnoDUALab_SDRTCA	605.6	−0.963	0.329	1892	−0.934	0.325
XnoDUALba_SDRTCA	590.0	−0.229	0.268	2087	−0.408	0.689

Note: Mann–Whitney U test. For all tests, the alternative hypothesis specifies that the control group is less than the depression group. XnoDUALab_ SDRTCA = standard deviation of reaction time of correct answers from block 2 of task XnoDUALab and correct responses from block 1 of task XnoDUALab; XnoDUALba_ SDRTCA = standard deviation of reaction time of correct answers from block 2 of task XnoDUALba and correct responses from block 1 of task XnoDUALba. * *p* < 0.05 ** *p* < 0.01.

## Data Availability

Data available on request due to privacy or ethical restrictions.
